# Predictive Contribution of Neutrophil/Lymphocyte Ratio in Diagnosis of Brucellosis

**DOI:** 10.1155/2015/210502

**Published:** 2015-02-03

**Authors:** Serdar Olt, Hasan Ergenç, Seyyid Bilal Açıkgöz

**Affiliations:** ^1^Department of Internal Medicine, Bitlis Mutki State Hospital, 13000 Bitlis, Turkey; ^2^Department of Internal Medicine, Sakarya University Medical Faculty Hospital, 54000 Sakarya, Turkey

## Abstract

Here we wanted to investigate predictive value of neutrophil/lymphocyte ratio (NLR) and platelet/lymphocyte ratio (PLR) in the diagnosis of brucellosis. Thirty-two brucellosis patients diagnosed with positive serum agglutination test and thirty-two randomized healthy subjects were enrolled in this study retrospectively. Result with ROC analyzes the baseline NLR and hemoglobin values were found to be significantly associated with brucellosis (*P* = 0.01, *P* = 0.01, resp.). Herein we demonstrated for the first time that NLR values were significantly associated with brucellosis. This situation can help clinicians during diagnosis of brucellosis.

## 1. Introduction

Brucellosis is a zoonotic disease that is transmittable to humans from infected animal reservoirs especially from milk and milk products [1]. Brucellosis may present with a broad spectrum of unspecific clinical manifestations, for example, fever, chills, sweating, malaise, arthralgia, weakness, back pain, and headache [2]. Many organ systems may be involved and it can be severe like endocarditis [3]. Most commonly serum agglutination test is used for diagnosis of brucellosis and its result conforms with complement fixation or Coombs' test [4]. In the diagnosis of brucellosis the gold standard test is culture of brucellosis [5]. Which patients must we suspect for brucellosis? In our clinic we suspect feverish patients with a history of milk and milk products intake. Laboratory and clinic findings are unclear in brucellosis patients. Because brucellosis patients show unspecific symptoms and unspecific laboratory findings, we aimed to investigate predictive contribution value of neutrophil/lymphocyte ratio (NLR) and platelet/lymphocyte ratio (PLR) in diagnosis of brucellosis.

## 2. Methods and Statistics

We collected clinical and laboratory data of thirty-two patients with brucellosis diagnosed with serum agglutination test and randomized thirty-two healthy people's data from hospital records retrospectively. We collected patients' blood samples at the admission and then we analyzed laboratory parameters that consist of haematological, biochemical, and serological tests. The white blood cell (WBC) and neutrophil and lymphocyte counts were recorded and NLR and PLR were calculated from these parameters. The cut-off titer value for serum agglutination test was 1/160 in diagnosis of brucellosis. We compare haematological parameters that consist of NLR and PLR between two groups.

All analyses were performed using SPSS for Windows (version 21.0; SPSS/IBM, Chicago, IL). Student's *t*-test, Pearson's chi-squared test, and logistic regression test were used when suitable. A *P* value <0.05 was considered statistically significant.

## 3. Results

The mean age of brucellosis patients was 41,7 ± 16,1. 62,5% of patients were male and 37,5% female, respectively. The most common brucellosis symptoms at the time of diagnosis were fatigue (37,5%), anorexia (34,4%), joint pain (34,4%), myalgia (31,3%), fever (25%), chills (18,8%), and feeling cold (18.8%), respectively (Table 1). We compare haematological parameters that consist of NLR and PLR between brucellosis patients and healthy groups. Result with ROC analyzes the baseline NLR and hemoglobin values were found to be significantly associated with brucellosis (*P* = 0.01, *P* = 0.01, resp.) (Table 2). There were no observed significant correlations with result of Fisher's exact test for NLR and hemoglobin values (Table 3). Result with Student's *t*-test, the PLR, neutrophil, lymphocyte, platelet count, WBC mean values was not significantly different between two groups (*P* > 0.05) (Table 4).

## 4. Discussion

We know that laboratory parameters and clinical symptoms are unspecific in brucellosis. High NLR values were found in inflammatory processes compared to noninflammatory processes in the literature [6–8].

NLR has been investigated for many diseases [9–16] but there are few studies between NLR and infectious diseases and there is not any study between brucellosis and NLR.

Therefore, we investigated predictive contribution value of NLR, PLR, and the other haematological parameters in diagnosis of brucellosis.

We compare NLR, PLR, and the other haematological parameters between brucellosis and healthy subjects. Result with ROC analyzes the baseline NLR and hemoglobin values were found to be significantly associated with brucellosis (*P* = 0.01, *P* = 0.01, resp.). There were no observed significant correlations with result of Fisher's exact test for NLR and hemoglobin values (*P* > 0.05). Result with Student's *t*-test, the PLR, neutrophil, lymphocyte, platelet count, WBC mean values was not significantly different between two groups (*P* > 0.05).

In a study conducted on patients with tuberculosis and sarcoidosis, Iliaz et al. demonstrated that the NLR was higher in patients with tuberculosis compared to the patients with sarcoidosis [17].

In a study conducted on patients with tuberculosis and bacterial community-acquired pneumonia, Yoon et al. showed that NLR levels were significantly lower in patients with pulmonary tuberculosis than in patients with bacterial community-acquired pneumonia [18].

In a study conducted on patients with Behçet disease, Rifaioglu et al. demonstrated that the NLR was higher in patients with active Behçet disease compared to controls and those with inactive Behçet disease [19].

In a study conducted on patients with Sjögren's syndrome, which is a chronic inflammatory disease, NLR was found significantly higher compared to the control group [20].

Additionally, in many studies on coronary artery disease, which is also an inflammatory process, a positive correlation was found for the NLR [21, 22].

In the literature high NLR values were found in inflammatory processes compared to control. In contrast with the literature data we found low NLR in brucellosis patients compared to control. In our study we demonstrated for the first time that NLR values were significantly associated with brucellosis.

Major limit of our study was the retrospective study that was considered. Single blood sampling was the other limitation of our study. For these reasons new prospectively controlled and randomized trials with multiple blood sampling must be performed to confirm our results. In spite of that we have suggested that low NLR may be useful data for brucellosis.

## Figures and Tables

**Table 1:** Symptoms and signs of patients with brucellosis at the admission.

	*n*	%

Fatigue	12	37,5
Anorexia	11	34,4
Joint pain	11	34,4
Myalgia	10	31,3
Sacroiliitis	8	25
Fever	6	18,8
Feeling cold	6	18,8
Splenomegaly	6	18,8
History of ingestion of fresh cheese	5	15,6
Chill	4	12,5
Hepatomegaly	3	9,4
Night sweats	3	9,4
Headache	3	9,4
Abdominal pain	2	6,3
Nausea	1	3,1
Vomiting	1	3,1
Constipation	1	3,1

**Table 2:** Model selection with ROC curve estimation in regression analysis for brucellosis.

Variable(s)	*P* value	95% confidence interval	
		Lower bound	Upper bound
WBC	0,50	0,30	0,59
Hemoglobin	**0,01**	0,19	0,45
Platelet	0,48	0,40	0,69
Neutrophil	0,07	0,23	0,51
Lymphocyte	0,31	0,43	0,71
PLR	0,95	0,35	0,64
NLR	**0,01**	0,19	0,46

Diagonal segments are produced by ties			

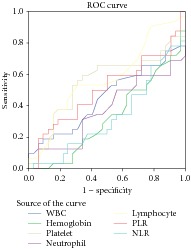			

**Table 3:** Comparison of haematological parameters between brucellosis and healthy group (Fisher's exact test).

Fisher's exact test		Number	*P* value
NLR < 1.5	Healthy group	21	0,06
	Brucellosis group	11	
NLR > 1.5	Healthy group	14	
	Brucellosis group	18	

Hemoglobin < 12.5	Healthy group	11	0,07
	Brucellosis group	21	
Hemoglobin > 12.5	Healthy group	5	
	Brucellosis group	27	

**Table 4:** Comparison of haematological parameters between brucellosis and healthy group (Student's *t*-test).

		Mean	SD	*P* value
PLR	Brucellosis group	108,31	41,61	0,93
	Healthy group	109,29	46,84	

Neutrophil	Healthy group	3640,62	1031,08	0,10
	Brucellosis group	3106,25	1356,21	

WBC	Brucellosis group	6187,50	1968,42	0,53
	Healthy group	6459,37	1163,65	

Platelet	Brucellosis group	242031,2	86953,63	0,67
	Healthy group	234281,2	62631,65	

Lymphocyte	Brucellosis group	2356,25	664,26	0,37
	Healthy group	2203,12	545,64	
